# Prognostic role of topoisomerase-II*α* in advanced ovarian cancer patients

**DOI:** 10.1038/sj.bjc.6604410

**Published:** 2008-05-27

**Authors:** G Ferrandina, M Petrillo, A Carbone, G Zannoni, E Martinelli, M Prisco, S Pignata, E Breda, A Savarese, G Scambia

**Affiliations:** 1 Department of Oncology, Catholic University, Campobasso, Italy; 2 Gynecologic Oncology Unit, Catholic University, Rome, Italy; 3 Institute of Human Pathology, Catholic University, Campobasso, Italy; 4 Institute of Human Pathology, Catholic University, Rome, Italy; 5 Medical Oncology, National Cancer Institute, Naples, Italy; 6 Medical Oncology, Ospedale S. Giovanni Calibita Fatebenefratelli, Rome, Italy; 7 Medical Oncology, Regina Elena Institute, Rome, Italy

**Keywords:** ovarian cancer, prognosis, topoisomerase-II*α*

## Abstract

To our knowledge, very few data about the role of Topoisomerase II*α* (TOPO-II*α*), an enzyme involved in critical steps of tumour cell proliferation and chemoresistance are currently available in ovarian cancer patients. The aim of this study was to investigate the prognostic value of TOPO-II*α* expression in a large, single institution series of 96 primary untreated advanced ovarian cancer patients admitted to the Gynecologic Oncology Unit, Catholic University of Campobasso and Rome. Immunohistochemistry was carried out by using the MoAb anti-human TOPO-II*α* antibody (clone Ki-S1). TOPO-II*α* immunoreaction was observed in 70 out of 96 cases (72.9%), and the percentages of positively stained cells ranged between 1 and 83% (median=10%). There was no association with clinico-pathological parameters. During the follow up period, progression and death of disease were observed in 76 (79.2%) and 45 (46.9%) cases. A statistically significant direct association between the percentages of positively immunostained tumour cells and the relative risk of death was observed (*χ*^2^=6.6, *P*-value=0.0101). In multivariate analysis, only platinum resistance, advanced stage of disease and high levels of TOPO-II*α* expression retained an independent negative prognostic role for OS. The unfavourable role of high TOPO-II*α* expression was maintained only in the subgroup of platinum resistant recurrent ovarian cancer patients, be TOPO-II*α* expression evaluated as continuous variable (*χ*^2^=5.1, *P*-value=0.024), or by means of the defined cutoff point. Our study suggests that the assessment of TOPO-II*α* could be helpful to identify poor prognosis platinum-resistant ovarian cancer patients, potentially candidates to investigational agents.

Despite the advances in surgical efforts, and the achievement of high response rates with platinum/paclitaxel front-line treatment (McGuire *et al*, 1996; Eisenkop *et al*, 2003; Ozols *et al*, 2003), ovarian cancer remains the most lethal gynaecological malignancy with a 5-year survival rate of 25–30% in advanced stage disease (Jemal *et al*, 2007). The major determinants of clinical outcome are represented by the extent of residual tumour at primary surgery, and sensitivity to platinum-based chemotherapy (Armstrong, 2007): Indeed, in platinum-resistant ovarian cancer patients, salvage chemotherapy with non-platinum agents mostly results in short-lived response rates, and poor survival (Armstrong, 2007). In this context, efforts aimed at identifying molecular factors eventually involved in chemotherapy resistance are actively ongoing. Several observations have recently suggested that Topoisomerase II*α* (TOPO-II*α*), one of the two isoforms an enzyme playing a relevant role in DNA replication, repair, and transcription (Chung *et al*, 1989), is involved in critical steps of tumour cell proliferation and chemoresistance (Wang, 2002). Strong TOPO-II*α* expression or enzymatic activity have been documented in ovarian carcinoma compared to the hardly detectable levels in benign ovarian tumours, ovarian inclusion cysts, and normal surface epithelium (van der Zee *et al*, 1991; Cornarotti *et al*, 1996; Withoff *et al*, 1999; Chekerov *et al*, 2006). The frequency of TOPO-II*α* overexpression in ovarian cancer has been reported to range between 30 and 70% (van der Zee *et al*, 1994; Gotlieb *et al*, 2001; Koshiyama *et al*, 2001), and a definite role of this enzyme as a marker of sensitivity not only to TOPO-II*α* targeting agents, such as anthracyclines and etoposide, but also to platinum agents *in vitro* and *in vivo* has been documented (Kikuchi *et al*, 1997; Naniwa *et al*, 2007).

In particular, a significant correlation between elevated TOPO-II*α* expression and tumour sensitivity to cisplatin-based chemotherapy has been shown in 37 primary untreated ovarian carcinomas (Cornarotti *et al*, 1996). While these observations favour the possibility that TOPO-II*α* overexpression could identify ovarian cancer patients with better clinical outcome, on the other hand other authors suggested that it might serve as a marker of aggressive features and poor prognosis (Gotlieb *et al*, 2001; Brustmann 2004; Mano *et al*, 2004). The discrepancies across earlier studies might be explained by the different design, the methodologies of TOPO-II*α* assessment, the small sample series, and the lack of data on salvage treatment (Cornarotti *et al*, 1996; Gotlieb *et al*, 2001; Brustmann 2004; Mano *et al*, 2004): indeed, it has to be taken into account that the analysis of the potential prognostic impact of TOPO-II*α* might be someway influenced by the role played by the same target as predictor of sensitivity to TOPO-II*α* inhibitory drugs, often used in the salvage setting.

To our knowledge, very few data about the role of TOPO-II*α* expression in predicting clinical outcome of ovarian cancer patients is currently available (Gotlieb *et al*, 2001; Brustmann 2004).

The aim of this study was to investigate the prognostic value of immunohistochemically assessed TOPO-II*α* expression in a large, single institution series of primary untreated advanced ovarian cancer patients.

## PATIENTS AND METHODS

### Patients

The study included 96 ovarian cancer patients admitted to the Gynecologic Oncology Unit, Catholic University of Campobasso and Rome. In our Institution a written informed consent is routinely requested to patients for collection of their clinical data, as well as paraffin embedded sections for research use. Clinico-pathological characteristics of the overall series are summarised in Table 1.

Median age was 60 years (range, 27–80). Seventy-seven cases (80.2%) were stage III and 19 (19.8%) cases were stage IV disease. According to the standard guidelines, maximal surgical effort has been attempted in all patients resulting in optimal debulking (residual tumour<1 cm) in 41 (42.7%) cases, which underwent surgical removal of tumour masses, along with total abdominal hysterectomy, adnexectomy, radical omentectomy appendectomy, multiple biopsies, and additional surgery (intestinal resections, diaphragm stripping) when required. Radical pelvic and para-aortic lymphadenectomy was performed in all patients undergoing primary cytoreduction who had residual tumour <1 cm. Suboptimal cytoreduction (residual tumour >1 cm) was achieved in 15 (15.6%) cases. Forty cases (41.7%) were judged to be unresectable at first surgery because of extensive peritoneal bulky carcinomatosis, agglutinated bowel/mesentery and infiltration of the upper gastrointestinal tract and /or the major vessels, and were submitted only to multiple biopsies. All patients received platinum-based chemotherapy (75–100 mg m^−2^ for cisplatin, AUC=5 for carboplatin, per cycle), including also paclitaxel (135–175 mg m^−2^ for each cycle) in 91 (94.8%) of cases. As far as patients undergoing only exploratory laparotomy are concerned, they received 3–4 cycles of chemotherapy before attempting a second cytoreductive surgery, unless they showed clinical progression during treatment. Response to chemotherapy was assessed according to WHO criteria (World Health Organization, 1979). In the subgroup of patients who were not susceptible to be cytoreduced at first surgery, a direct assessment of the extent of response to chemotherapy was carried out at time of second laparotomy. At recurrence/progression of disease platinum sensitive patients were triaged to platinum/taxane-containing regimen, while platinum-resistant patients were administered pegylated liposomal doxorubicin (PLD) according to clinical trials ongoing in our Institution (Ferrandina *et al*, 2007, 2008).

### Immunohistochemistry

Pretreatment tumour tissues biopsies were obtained at first surgery in all cases. Tissue specimens were fixed in 10% formalin and paraffin-embedded according to standard procedures. Immunostaining was performed on 3 *μ*m tissue sections mounted on poly-l-lysine-coated slides and dried at 37°C overnight. After the slides were deparaffinised in xylene, and rehydrated conventionally, the endogenous peroxidase activity was blocked with 3% H_2_O_2_ in TBS for 5 min. Antigen retrieval procedure was performed by microwave oven heating in citrate buffer (pH=6). Sections were incubated with 20% normal goat serum for 30 min at room temperature to reduce nonspecific binding, then with the monoclonal mouse anti-human TOPO-II*α* antibody (clone Ki-S1) (diluted 1 : 50) (Dako Cytomation, Denmark) in 20% goat serum. TOPO-II*α* detection was evaluated by a labelled polymer The En Vision-mouse + System-HRP System (DAKO, Carpinteria, CA, USA) was used. Diaminobenzidine was used as a chromogen (DAB substrate System, DAKO). Positive controls for TOPO-II*α* was represented by sections taken from the breast. Results were expressed as the proportion of immunostained tumour cells. The analysis of all tissue sections was done without any prior knowledge of the clinical parameters by two authors (AC, GFZ) by means of light microscopy. The proportion of immunostained tumour cells was scored at low magnification (× 5 objective lens) by evaluating the entire tumour area. The accuracy of immunohistochemical readings was evaluated by assessing intra-and inter-observer variability (mean±s.d.=8%±2, and 12%±3, respectively).

### Statistical analysis

Wilcoxon signed ran sum test was used to analyse the expression levels of TOPO-II*α* according to clinico-pathological parameters. Time to progression and overall survival (OS) were calculated from the date of diagnosis to the date of progression/death or date last seen. Medians and life tables were computed using the product-limit estimate by the Kaplan and Meyer (1958) method and the log–rank test was employed to assess the statistical significance (Mantel, 1966). Statistical analysis was carried out using SOLO (BMDP Statistical Software, Los Angeles, CA, USA). Multivariate analysis assessing the clinical role of TOPO-II*α* expression matched with other clinico-pathological characteristics was performed by Cox's proportional hazards model (Cox, 1972).

## RESULTS

**Figure 1:**
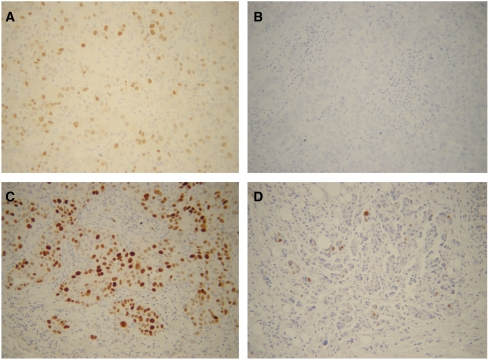
TOPO-II*α* immunoreaction in primary ovarian cancer. (**A**) Positive control (human breast cancer tissue specimen), (**B**) negative control (ovarian carcinoma) for TOPO-II*α* staining. Representative examples of high (**C**) and low (**D**) TOPO-II*α* expression. (**A**, **B**, **C**, **D**) Magnification= × 200.

Figure 1 shows representative examples of high *vs* low TOPO-II*α* expression in primary ovarian cancer. Specific immunostaining for TOPO-II*α* was exclusively confined to the nuclei, and showed a wide range of variability: in the overall series, TOPO-II*α* immunoreaction was observed in 70 out of 96 cases (72.9%), and the percentages of positively stained cells ranged between 1 and 83% (median=10%). Given the large inter-tumour variability, the absence of a defined scoring system, and the need to minimise any source of bias related to the use of a specific cutoff value, analysis of the data was carried out by using the values of TOPO-II*α* as a continuous variable.

The percentages of TOPO-II*α* immunoreactive tumour cells were found not to be associated with any of the clinico-pathological parameters examined. Moreover, no association with response to first-line treatment was documented (Table 1).

Follow-up data were available for all patients. As of December 2007, the median follow up was 37 months (range, 6–120). During the follow up period, progression and death of disease were observed in 76 (79.2%) and 45 (46.9%) cases.

**Figure 2:**
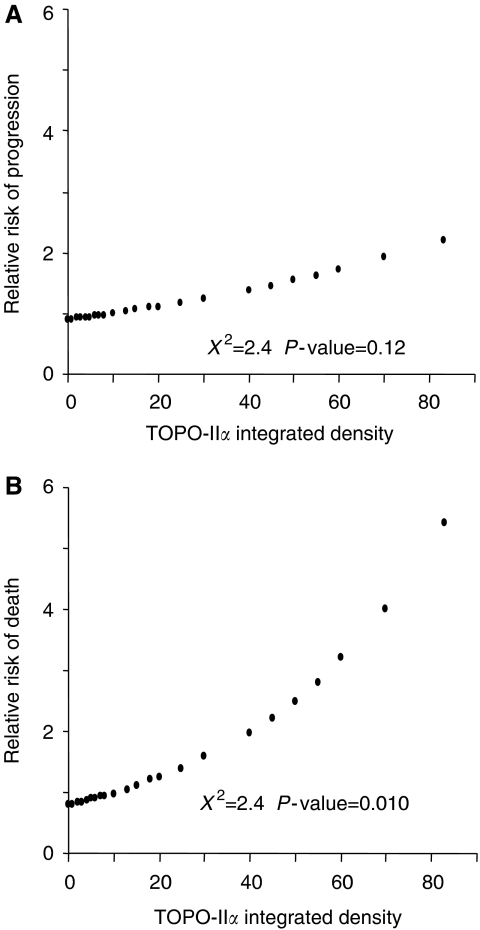
Plot of the estimates of the relative risk of progression (**A**) and death (**B**) of disease as a prediction of TOPO-II*α* values, calculated by COX's proportional hazard regression model.

Figure 2 shows the plot of the estimates of the relative risk of progression or death as a prediction of TOPO-II*α* values, calculated by COX's proportional hazard regression model: there was no association between the percentage values of positively TOPO-II*α* immunostained tumour cells and the relative risk of progression of disease (*χ*^2^=2.3, *P*-value=0.12).

On the other hand, a statistically significant direct association between the percentages of TOPO-II*α* positively immunostained tumour cells and the relative risk of death was observed (*χ*^2^=6.6, *P*-value=0.0101) (see also Table 2).

**Figure 3:**
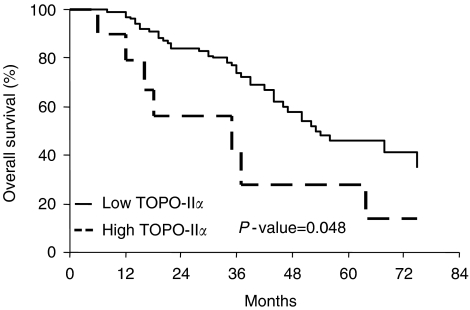
Overall survival curves in ovarian cancer patients according to the status of TOPO-II*α*.

We were then prompted at defining the cutoff value of TOPO-II*α* that more closely correlated with the risk of death: the most significant association was observed at the cutoff value of 25% TOPO-II*α* immunoreactive cells: cases with high TOPO-II*α* expression has a shorter OS (median OS=35 months) than cases with low TOPO-II*α* levels (median=54 months) (*P*-value=0.048) (Figure 3).

In univariate analysis of OS, platinum resistance, more advanced stage of disease, and suboptimal residual tumour at primary surgery were also found to be associated with a high risk of death of disease (Table 2). In multivariate analysis, only platinum resistance, more advanced stage of disease and high levels of TOPO-II*α* expression retained an independent negative prognostic role for OS (Table 2).

We were then prompted at analyzing the prognostic relevance of TOPO-II*α* expression in platinum-sensitive *vs* platinum-resistant ovarian cancer patients: interestingly enough, the unfavourable role of high TOPO-II*α* expression was maintained only in the subgroup of platinum-resistant recurrent ovarian cancer patients, be it evaluated as continuous variable (*χ*^2^=5.1, *P*-value=0.024), or by means of the defined cutoff point: cases with high TOPO-II*α* expression has a shorter OS (median OS=18 months) than cases with low TOPO-II*α* levels (median=35 months) (*P*-value=0.041) (data not shown).

## DISCUSSION

This is the first study analysing the association between the expression of TOPO-II*α* protein and clinical outcome in a large series of primary untreated ovarian cancer patients.

We showed that patients whose tumours express high levels of TOPO-II*α* experience a shorter OS compared to cases with low TOPO-II*α* content, as also suggested by preliminary studies (Gotlieb *et al*, 2001; Mano *et al*, 2004).

The analysis of the percentages of positively TOPO-II*α* immunostained cells as a continuous value allowed to avoid the potential bias inherent in the use of an arbitrary cutoff point, and further supported the association of high TOPO-II*α* expression with a high risk of death of disease.

The independent role of high TOPO-II*α* expression as marker of poor prognosis is sustained by the lack of association with any of the clinico-pathological parameters examined, and also by the results of multivariate analysis documenting the persistence of the unfavourable significance of high TOPO-II*α* expression after adjusting for stage of disease, residual tumour, and platinum responsiveness.

The association between TOPO-II*α* overexpression and poor prognosis has been reported in other human tumours, including breast and bladder carcinomas (Kruger *et al*, 2005; O'Connor *et al*, 2006) as well as glioblastoma and lymphoma (Ho *et al*, 2003; Schrader *et al*, 2004); however, the assessment of whether the prognostic impact of this parameter is due to its value as predictor of response to treatment-based regimen or as a marker of intrinsic tumour aggressiveness (pure prognostic factor), is difficult to be established. Indeed, the data about the role of TOPO-II*α* in determining susceptibility to platinum agents are very controversial since increased levels of TOPO-II*α* have been found both in cell lines or small subsets of patients resistant to alkylating agents or platinum drugs (de Jong *et al*, 1990; Chu, 1994; Kikuchi *et al*, 1997), as well as in *in vitro* models and ovarian cancer patients exhibiting sensitivity to cisplatin (Giaccone *et al*, 1992; Cornarotti *et al*, 1996; Koshiyama *et al*, 2001). In our series, we could not detect any difference in the distribution of TOPO-II*α* values according to response to first line chemotherapy, or time to progression, the latter one being strictly associated with treatment susceptibility, thus suggesting that overexpression of TOPO-II*α* might more likely indicate tumour-intrinsic biological aggressiveness, rather than represent only a marker of platinum sensitivity. In this context, it is noteworthy that TOPO-II*α* is involved in several biological pathways of tumour aggressiveness: for instance, TOPO-II*α* is correlated with the proliferation associated marker ki67 (Costa *et al*, 2000), the Vascular Endothelial Growth Factor (Brustmann, 2004), and also with the product of erbB2/neu oncogene (Mano *et al*, 2004; Hicks *et al*, 2005); moreover, TOPO-II*α* expression seems to be regulated by p53 oncosuppressor gene (Sandri *et al*, 1996), whose mutations are differentially involved in platinum *vs* paclitaxel sensitivity in ovarian cancer (Ferrandina *et al*, 1999; Lavarino *et al*, 2000). Interestingly enough, the unfavourable prognostic role of high TOPO-II*α* content was documented only in the subgroup of platinum-resistant ovarian cancer patients. This finding, which cannot be ascribed to differences in the expression levels of TOPO-II*α* according to platinum sensitivity, raises some issues: (i) the availability of a molecular factor able to discriminate high- *vs* low-risk patients in the platinum-resistant subgroup seems clinically relevant, when considering that the prognosis of patients defined as platinum-resistant is so unfavourable, that any other factor is expected to become minor, if any, importance in terms of prognostic discrimination; (ii) considering that in our series all platinum-resistant recurrent ovarian cancer patients were administered doxorubicin, our findings seem to demonstrate that the role of TOPO-II*α* as an unfavourable prognostic factor might overcome its role as a predictor of response to TOPO-II*α*-targeting agents: the discrepancy with *in vitro* data (Koshiyama *et al*, 2001) has to be considered in the light of the common experience that tissue specimens represent a different picture from ‘*in vitro*’ models: the contribution of inflammatory and endothelial cells in the stroma, is likely to realise a regulatory microenvironment which might somehow hide the straight biochemical relationships found in cell cultures. In this context, recent observations have been provided (Chekerov *et al*, 2006) that the pattern of expression of TOPO-II*α* in ovarian cancer varies in epithelial *vs* adjacent stromal cell component. It remains to be clarified why TOPO-II*α* expression has definitely a strong clinical impact only in the subgroup of platinum-resistant ovarian cancer patients: it is conceivable that a specific molecular profile might characterise platinum-resistant ovarian carcinomas compared to platinum-sensitive ones, so that only in this subset of patients TOPO-II*α* might be coexpressed with molecular factor(s) eventually involved in determining more aggressive tumour features. These observations emphasize the need to proceed to a multiparametric evaluation of several biologically linked molecular targets, to possibly define clusters of patients with a more aggressive tumour ‘molecular profile’.

In conclusion, although these findings need to be confirmed in a larger series, our study suggests that the assessment of TOPO-II*α* could be helpful to identify poor prognosis platinum resistant ovarian cancer patients, potentially candidates to investigational agents.

## Figures and Tables

**Table 1:** Clinico-pathological characteristics of the overall series, and TOPO-II*α* expression

		**Percentage of TOPO-II*α* positive cells**	
**Characteristics**	**No. of patients (%)**	**Median (range)**	***P*-value^a^**
All cases	96	5 (0–83)	
			
*Age (years)*			
⩽65	31 (32.3)	4 (0–50)	
>65	65 (67.7)	5 (0–83)	0.4
			
*FIGO Stage*			
III	77 (80.2)	5 (0–83)	
IV	19 (19.8)	3 (0–50)	0.5
			
*Grade*			
G1-2	18 (18.7)	5 (0–60)	
G3	67 (69.8)	5 (0–83)	0.8
n.a.	11		
			
*Histotype*			
Serous	84 (87.5)	5 (0–83)	
Other	12 (12.5)	4 (0–50)	0.6
			
*Residual tumour*			
<1 cm	41 (42.7)	4 (0–60)	
>1 cm	15 (15.6)	4 (0–40)	
Exploratory laparotomy	40 (41.7)	7 (0–83)	0.7^b^
			
*Primary chemotherapy*			
Platinum/paclitaxel	91 (94.8)	5 (0–83)	
Platinum-based	5 (5.2)	5 (0–18)	0.8
			
*Response to CT*			
Yes	48 (50.0)	4.5 (0–60)	
No	48 (50.0)	5 (0–83)	0.8

n.a.=not available.

a Calculated by Mann–Whitney nonparametric test.

b Calculated by Kruskall–Wallis sum test.

**Table 2:** Univariate and multivariate analysis of clinico-pathological parameters and TOPO-II*α* as prognostic factors for overall survival in advanced ovarian cancer patients

	**Univariate**			**Multivariate^a^**		
**Variable**	**RR1**	***χ* ^2^**	***P*-value**	**RR2**	***χ* ^2^**	***P*-value**
*Age (years)*						
<65	1^0^			—	—	—
>65	1.2	0.3	0.6			
						
*Stage*						
III	1^0^			1^0^		
IV	3.0	9.4	0.0021	1.96	7.1	0.008
						
*Extent of residual tumour*						
<1 cm	1^0^			1^0^		
>1 cm	3.1	9.5	0.0020	1.63	1.5	0.22
						
*Response to treatment*						
No	1^0^			1^0^		
Yes	9.7	35.0	0.0001	8.2	30.2	0.0001
						
*Topo-IIα percentages*						
	1^0^			1^0^		
Continuous data^b^	1.02^b^	6.6	0.0101	1.02^b^	3.9	0.0477

1^0^=Reference category.

*χ*^2^ of the model=45.7; *P*-value=0.0001.

a Only variables with *P*-value<0.20 in the univariate analysis were included in the multivariate model, RR1=unadjusted relative risk, RR2=relative risk after adjusting for all the factors listed

b Relative risk per percentage unit of Topo-II*α* increase.
